# hERG1 channel subunit composition mediates proton inhibition of rapid delayed rectifier potassium current (*I*_Kr_) in cardiomyocytes derived from hiPSCs

**DOI:** 10.1016/j.jbc.2022.102778

**Published:** 2022-12-08

**Authors:** Chiamaka U. Ukachukwu, Eric N. Jimenez-Vazquez, Abhilasha Jain, David K. Jones

**Affiliations:** 1 Department of Pharmacology, University of Michigan Medical School, Ann Arbor, Michigan, USA; 2 Department of Internal Medicine, University of Michigan Medical School

**Keywords:** acidosis, hERG, *I*_Kr_, iPSC-CMs, hERG1a, hERG1b, PAS domain, AP, action potential, CHO, Chinese hamster ovary cell line, CM, cardiomyocyte, hiPSC-CM, human-induced pluripotent stem cell–derived cardiomyocyte, *I*_Kr_, rectifier potassium current, LQTS, long QT syndrome, mERG1b, mouse ERG1b, PAS, Per–Arnt–Sim, PDMS, polydimethylsiloxane, RMP, resting membrane potential, SIDS, sudden infant death syndrome

## Abstract

The voltage-gated channel, hERG1, conducts the rapid delayed rectifier potassium current (*I*_Kr_) and is critical for human cardiac repolarization. Reduced *I*_Kr_ causes long QT syndrome and increases the risk for cardiac arrhythmia and sudden death. At least two subunits form functional hERG1 channels, hERG1a and hERG1b. Changes in hERG1a/1b abundance modulate *I*_Kr_ kinetics, magnitude, and drug sensitivity. Studies from native cardiac tissue suggest that hERG1 subunit abundance is dynamically regulated, but the impact of altered subunit abundance on *I*_Kr_ and its response to external stressors is not well understood. Here, we used a substrate-driven human-induced pluripotent stem cell–derived cardiomyocyte (hiPSC-CM) maturation model to investigate how changes in relative hERG1a/1b subunit abundance impact the response of native *I*_Kr_ to extracellular acidosis, a known component of ischemic heart disease and sudden infant death syndrome. *I*_Kr_ recorded from immatured hiPSC-CMs displays a 2-fold greater inhibition by extracellular acidosis (pH 6.3) compared with matured hiPSC-CMs. Quantitative RT-PCR and immunocytochemistry demonstrated that hERG1a subunit mRNA and protein were upregulated and hERG1b subunit mRNA and protein were downregulated in matured hiPSC-CMs compared with immatured hiPSC-CMs. The shift in subunit abundance in matured hiPSC-CMs was accompanied by increased *I*_Kr_. Silencing hERG1b’s impact on native *I*_Kr_ kinetics by overexpressing a polypeptide identical to the hERG1a N-terminal Per-Arnt-Sim domain reduced the magnitude of *I*_Kr_ proton inhibition in immatured hiPSC-CMs to levels comparable to those observed in matured hiPSC-CMs. These data demonstrate that hERG1 subunit abundance is dynamically regulated and determines *I*_Kr_ proton sensitivity in hiPSC-CMs.

hERG1, encoded by *KCNH2*, is the voltage-gated potassium channel that conducts the rapid delayed rectifier potassium current (*I*_Kr_). Reduced *I*_Kr_ from either off-target pharmacological block or loss-of-function *KCNH2* variants causes the cardiac disorder long QT syndrome (LQTS) and increases the risk for cardiac arrhythmia, syncope, and sudden cardiac death (1, 2). LQTS is the leading cause of arrhythmic death in children and accounts for 5 to 10% of sudden infant death syndrome (SIDS) and intrauterine fetal death cases (3, 4, 5, 6, 7, 8). Furthermore, multiple LQTS-associated *KCNH2* variants have been linked with intrauterine fetal death and SIDS, underscoring the importance of hERG1 in the young heart (3, 9, 10, 11, 12, 13).

Native heRG1 channels comprise at least two subunits, hERG1a and hERG1b (14, 15, 16, 17, 18). Mutations in both subunits promote/cause cardiac electrical dysfunction (3, 19, 20, 21). hERG1a subunits contain an N-terminal Per–Arnt–Sim (PAS) domain that regulates channel gating through interactions with the C-terminal cyclic nucleotide–binding homology domain and the cytoplasmic S4–S5 linker (20, 22, 23, 24, 25). hERG1b has a much shorter and unique N terminus that lacks a PAS domain (14, 15). When heterologously expressed in human embryonic kidney 293 cells, the absence of a functional PAS domain in hERG1b triggers a roughly twofold acceleration in the time course of activation, deactivation, and inactivation recovery in heteromeric hERG1a/1b channels compared with homomeric hERG1a channels (20). In human-induced pluripotent stem cell–derived cardiomyocyes (hiPSC-CMs), silencing hERG1b by overexpressing a polypeptide that mimics the hERG1a PAS domain slows native *I*_Kr_ gating kinetics and reduces *I*_Kr_ magnitude, triggering increased action potential (AP) duration and early afterdepolarizations (16). Conversely, disabling the hERG1a PAS domain using PAS-targeting antibodies accelerates *I*_Kr_ gating, increases *I*_Kr_ magnitude, and hastens cardiac repolarization (26).

Extracellular acidosis is a major inhibitor of *I*_Kr_ (27) and occurs in a variety of pathological situations associated with cardiac dysfunction, including SIDS and myocardial ischemia (28, 29, 30). Consequently, a large body of work has explored the impact of extracellular protons on hERG1 (27, 31, 32, 33, 34, 35, 36, 37). Briefly, reduced extracellular pH reduces hERG1 channel conductance, depolarizes channel voltage dependence of activation, and accelerates channel deactivation (31, 33, 34, 38). The proarrhythmic effects of reduced pH on hERG1 are twofold, pore block by protons slows cardiac repolarization, whereas the accelerated deactivation impairs the ability of hERG1 to protect the heart from premature stimulation (27, 39, 40). Interestingly, it was demonstrated that the inhibitory effect of extracellular protons is enhanced in hERG1b-containing channels (41, 42).

Several studies suggest that hERG1 subunit abundance is dynamically regulated *in vivo* (18, 43, 44, 45, 46, 47, 48). However, the mechanisms that determine hERG1 subunit abundance and the impact of altered subunit abundance on the susceptibility of arrhythmia are poorly understood. LQTS mutations in the hERG1a PAS domain were shown to disrupt hERG1b trafficking to the membrane (49). In murine tissue, targeted mouse ERG1b (mERG1b) deletion not only abolishes *I*_Kr_ in adult mice but also reduces *I*_Kr_ magnitude by roughly 50% in neonates, compared with wildtype littermate controls (50). These data suggest that mERG1a is selectively downregulated during maturation of the murine heart. In the human heart, hERG1a mRNA transcripts are upregulated and hERG1b transcripts are downregulated in adult ventricular tissue compared with fetal cardiac tissue (3). Similarly, the relative abundance of hERG1a to hERG1b protein was reduced in failing cardiac tissue compared with nondiseased donor controls (51).

In this study, we used *in vitro* maturation of hiPSC-CMs to probe the impact of hERG1 subunit dynamics on proton modulation of native cardiac *I*_Kr_. The data presented herein demonstrate that increased hERG1a and reduced hERG1b in matured hiPSC-CMs diminish *I*_Kr_ sensitivity to extracellular protons compared with *I*_Kr_ recorded from immatured hiPSC-CMs.

## Results

Protons decrease hERG1 current amplitude and accelerate the time course of hERG1 deactivation (27, 31, 33, 34, 37, 52). However, the specific effects of extracellular acidosis can vary across expression systems. For example, the impact of protons on the voltage dependence of activation is not consistently reported. These variations across systems suggest that other unidentified factors contribute to the response of hERG1 to protons (33, 35, 36, 37, 39, 52, 53). Subunit abundance is one factor that may explain the different acidosis sensitivities. To determine the impact of hERG1 subunit abundance on native *I*_Kr_ sensitivity, we cultured hiPSC-CMs on two different matrices to promote distinct stages of maturation and corresponding shifts in hERG1 subunit expression.

### Extracellular matrix mediates hiPSC-CM maturation

**Figure 1:**
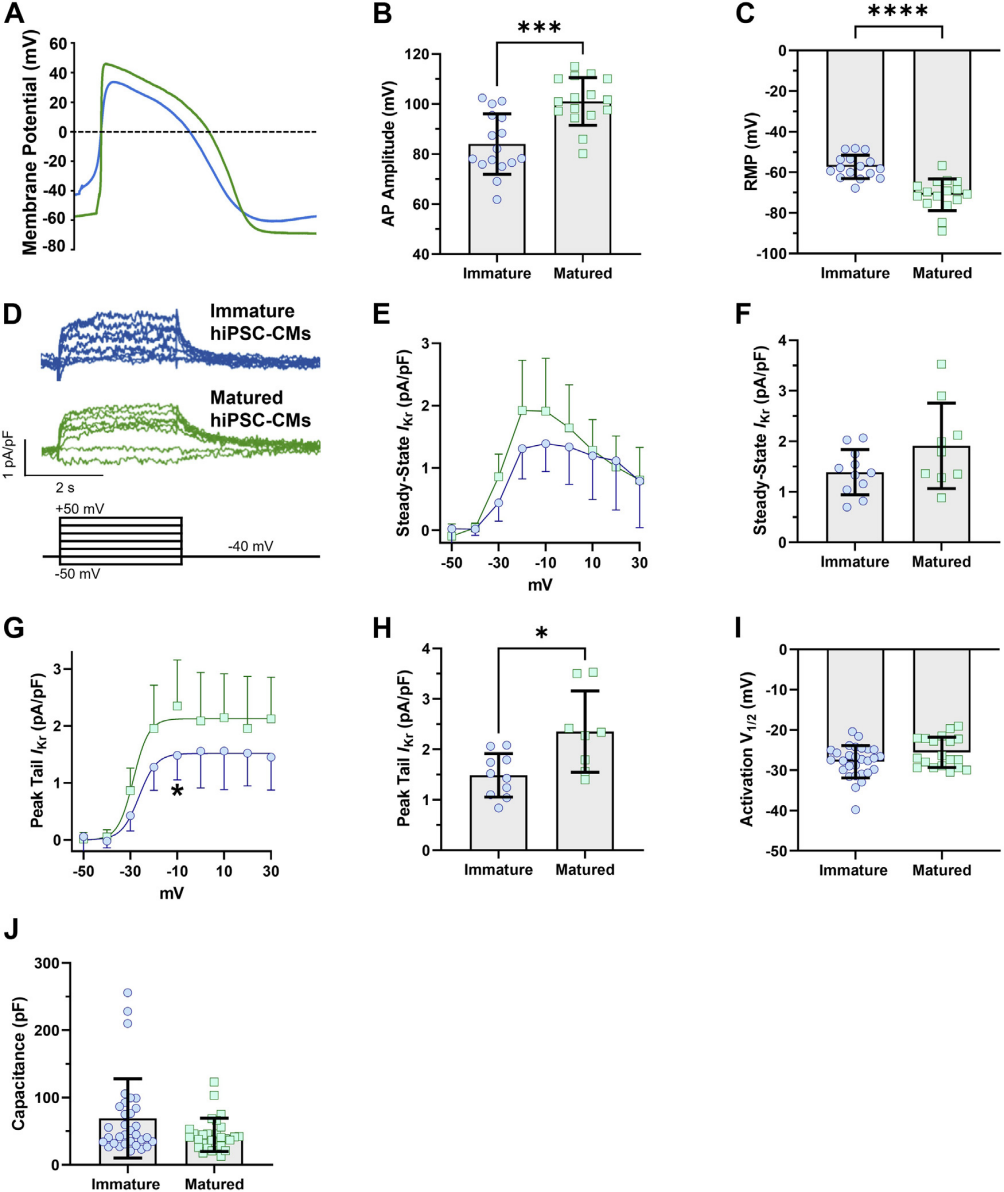
**hiPSC-CM maturation with PDMS hyperpolarizes the AP and increases *I***_**Kr**_**density.***A*, AP recordings from cells cultured on glass (*blue*) and PDMS (*green*). *B* and *C*, AP parameters. Cells plated on PDMS demonstrated more hyperpolarized resting membrane potential and greater AP amplitude than cells plated on glass. *D*, representative *I*_Kr_ traces elicited by the protocol at *bottom*. *E*, steady-state *I*_Kr_ measured at the end of the step pulse, recorded from immatured and matured hiPSC-CMs. *F*, steady-state current densities at −10 mV. *G*, tail *I*_Kr_ in immatured and matured hiPSC-CMs. *H*, tail current densities at −10 mV. hiPSC-CMs plated on PDMS had larger ERG currents than hiPSC-CMs plated on glass. *I*, there was no significant difference in the voltage dependence (*V*_1/2_) of *I*_Kr_ activation recorded from immatured hiPSC-CMs *versus* matured hiPSC-CMs. *J*, there was no statistically significant difference in the cellular capacitance between immatured and matured hiPSC-CMs. Data were compared using a two-way ANOVA test and a two-tailed Mann–Whitney test where appropriate. Errors bars represent mean ± SD. N value = 3, n value ≥8. ∗∗∗∗*p* < 0.0001, ∗∗∗*p* = 0.0002, and ∗*p* < 0.05. AP, action potential; hiPSC-CM, human-induced pluripotent stem cell–derived cardiomyocyte; *I*_Kr_, rectifier potassium current; PDMS, polydimethylsiloxane.

Culturing hiPSC-CMs on a pliable substrate promotes hiPSC-CM maturation, although the “matured” hiPSC-CMs still retain features of immaturity including irregular shape and absence of t-tubules (54, 55, 56). We cultured hiPSC-CMs on either a pliable substrate (polydimethylsiloxane [PDMS]) or a stiff substrate (glass). All substrates were coated with Matrigel prior to hiPSC-CM plating. In previous reports using PDMS as a substrate, hiPSC-CMs have more mature electrophysiological features (*e.g.*, increased *I*_Na_ and *I*_K1_, faster upstroke velocity and faster conduction velocity, hyperpolarized resting membrane potential [RMP], etc.) compared with hiPSC-CMs plated on a hard substrate (54). Here, we found that hiPSC-CMs cultured on Matrigel-coated PDMS displayed electrophysiological characteristics consistent with enhanced maturation compared with hiPSC-CMs cultured on Matrigel-coated glass coverslips (Fig. 1). APs recorded from hiPSC-CMs cultured on PDMS displayed hyperpolarized RMPs and larger AP amplitudes compared with APs recorded from hiPSC-CMs cultured on glass (Table 1 and Fig. 1, *A*–*C*). In addition, E-4031-sensitive currents, which are indicative of native *I*_Kr_, showed a trend to be increased in PDMS-cultured hiPSC-CMs compared with glass-cultured hiPSC-CMs. Steady-state *I*_Kr_ density, measured at the end of a 3 s step pulse, was increased from 1.3 ± 0.1 pA/pF in glass-cultured hiPSC-CMs to 1.9 ± 0.3 pA/pF in PDMS-cultured hiPSC-CMs (Table 1 and Fig. 1, *D*–*F*). Tail *I*_Kr_ was similarly increased, from 1.4 ± 0.1 pA/pF in glass-cultured hiPSC-CMs to 2.3 ± 0.3 pA/pF in PDMS-cultured hiPSC-CMs (Table 1 and Fig. 1, *D*, *G*, and *H*). hiPSC-CM maturation had no effect on the voltage dependence of *I*_Kr_ activation (Fig. 1*I*). There was no significant difference in cell capacitance between immatured and matured cells (Fig. 1*J*).

**Table 1:** Biophysical parameters of immatured and matured hiPSC-CMs

Biophysical parameter		Immatured hiPSC-CMs	SD	n	Matured hiPSC-CMs	SD	n
APA (mV)		84^a^	12.08	16	101.03	9.54	16
RMP (mV)		−57.29^b^	5.75	16	−71.04	7.86	16
Steady-state *I*_Kr_ at −10 mV (pA/pF)		1.39	0.44	11	1.91	0.84	9
Peak tail *I*_Kr_ at −10 mV (pA/pF)		1.48^c^	0.43	10	2.35	0.8	8
Steady-state *I*_Kr_ at −10 mV (pA/pF)	pH 7.4	1.47^d^	0.47	7	1.91^a^	0.84	10
	pH 6.3	0.3^c^	0.27	6	0.93	0.52	10
Peak tail *I*_Kr_ at −10 mV (pA/pF)	pH 7.4	0.78^c^	0.61	8	1.61^d^	0.61	10
	pH 6.3	0.36	0.4	7	0.65	0.4	10
Relative steady-state *I*_Kr_ at −10 mV (*I*_SS_/*I*_SSMAX_)	pH 7.4	0.9^a^	0.22	7	0.96^a^	0.1	10
	pH 6.3	0.38^c^	0.15	6	0.51	0.25	10
Relative peak tail *I*_Kr_ at −10 mV (*I*_Tail_/*I*_TailMAX_)	pH 7.4	0.95^d^	0.07	8	0.9^b^	0.06	10
	pH 6.3	0.34	0.14	7	0.33	0.14	10
*V*_1/2_ (mV)	pH 7.4	−26.74^d^	5.95	7	−22.58^a^	2.57	10
	pH 6.3	−15.39	3.92	7	−13.88	6.13	10
Fast Tau (ms)	pH 7.4	117.04^a^	79.84	12	194.83^c^	249.35	12
	pH 6.3	40.96	36.82	12	80.45	122.93	12

Abbreviation: APA, action potential amplitude.

Two-tailed Mann–Whitney test. N value = 3 and n value ≥6.

a *p* = 0.0024.

b *p* < 0.0001.

c *p* < 0.05.

d *p* ≤ 0.0008.

**Figure 2:**
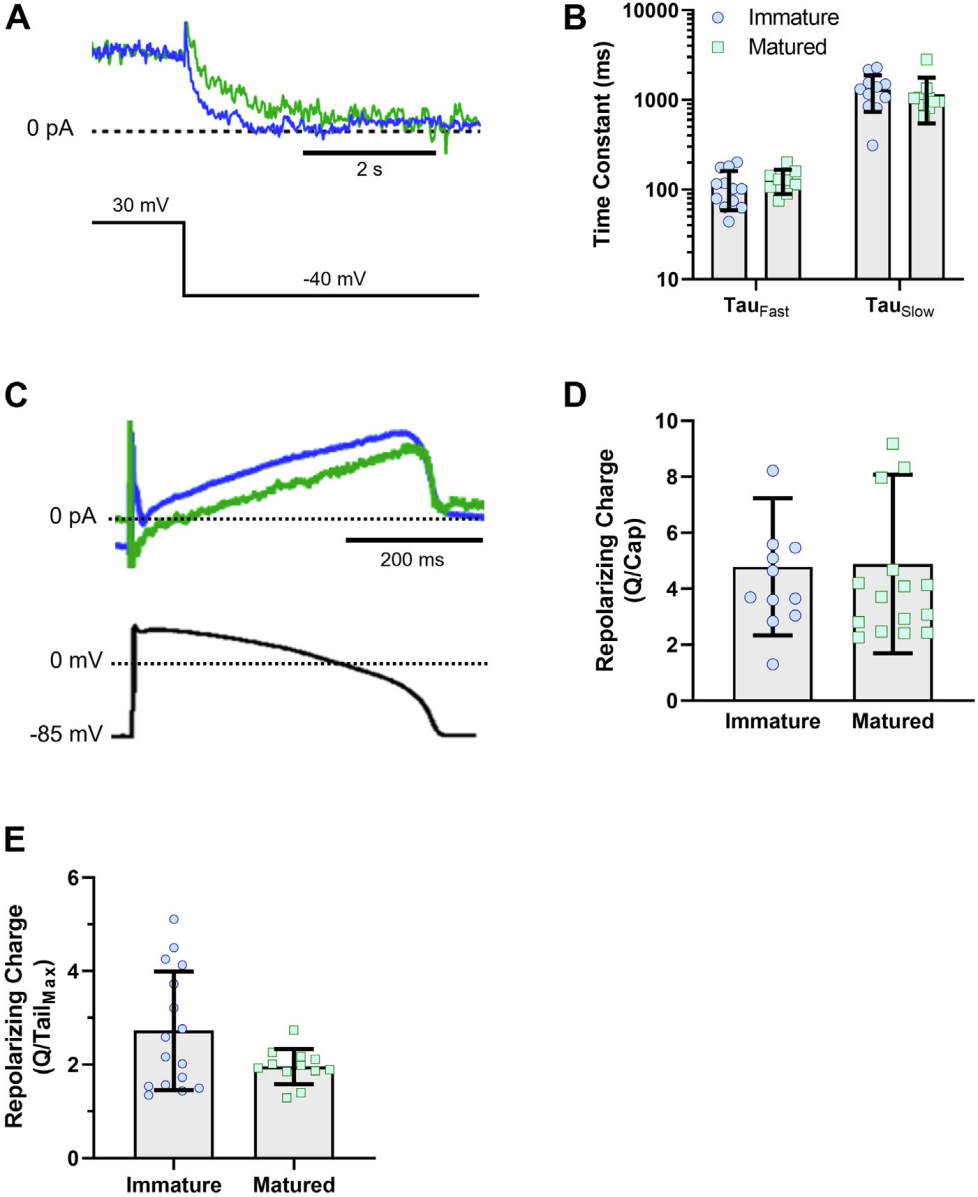
**Effect of cell maturation on *I***_**Kr**_**deactivation.***A*, tail *I*_Kr_ traces recorded from immatured (*blue*) and matured (*green*) hiPSC-CMs at −40 mV. *B*, corresponding deactivation time constants for *I*_Kr_ recorded from hiPSC-CMs cultured on glass (immatured) *versus* PDMS (matured). *C*, representative *I*_Kr_ traces recorded from immatured (*blue*) and matured (*green*) hiPSC-CMs using a ventricular AP clamp protocol. *D*, repolarizing charge normalized to the cell capacitance in matured and immatured hiPSC-CMs. *E*, repolarizing charge normalized to the peak-tail *I*_Kr_ of the same cell recorded from matured and immatured hiPSC-CMs. Two-tailed Mann–Whitney test. Errors bars represent mean ± SD. N value = 3, n value ≥10. AP, action potential; hiPSC-CM, human-induced pluripotent stem cell–derived cardiomyocyte; *I*_Kr_, rectifier potassium current; PDMS, polydimethylsiloxane.

We also investigated the impact of hiPSC-CM maturation on *I*_Kr_ kinetics. We fit the decay of tail currents at −40 mV with a biexponential equation (Equation 2). The fits yielded fast (τ_fast_) and slow (τ_slow_) time constants that were similar in matured (118.4 ± 10 ms and 1173 ± 216 ms for τ_fast_ and τ_slow_, respectively) compared with immatured hiPSC-CMs (110 ± 15 ms and 1313.5 ± 174 ms for τ_fast_ and τ_slow_, respectively) (Fig. 2, *A* and *B*). We also recorded *I*_Kr_ during a voltage command designed to mimic a human ventricular AP (Fig. 2*C*). We integrated E-4031-sensitive currents elicited during the AP waveform and normalized the resultant charge to cell capacitance. Surprisingly, despite the substantial increase in tail *I*_Kr_ density, there was no significant difference in *I*_Kr_ charge densities recorded from immatured and matured hiPSC-CMs (Fig. 2*D*). To test if additional changes in *I*_Kr_ kinetics could be present in matured hiPSC-CMs, we normalized the *I*_Kr_ charge recorded during the AP waveform to the peak tail *I*_Kr_ recorded from the same cell. Like *I*_Kr_ deactivation, relative repolarizing charge in matured hiPSC-CMs trended to a reduction compared with immatured hiPSC-CMs, but the difference was not statistically significant (*p* = 0.22, Fig. 2*E*). This may indicate differences in gating kinetics, where channel activation is slowed or inactivation is stabilized.

### External acidosis differentially impacts *I*_Kr_ recorded from matured and immatured hiPSC-CMs

**Figure 3:**
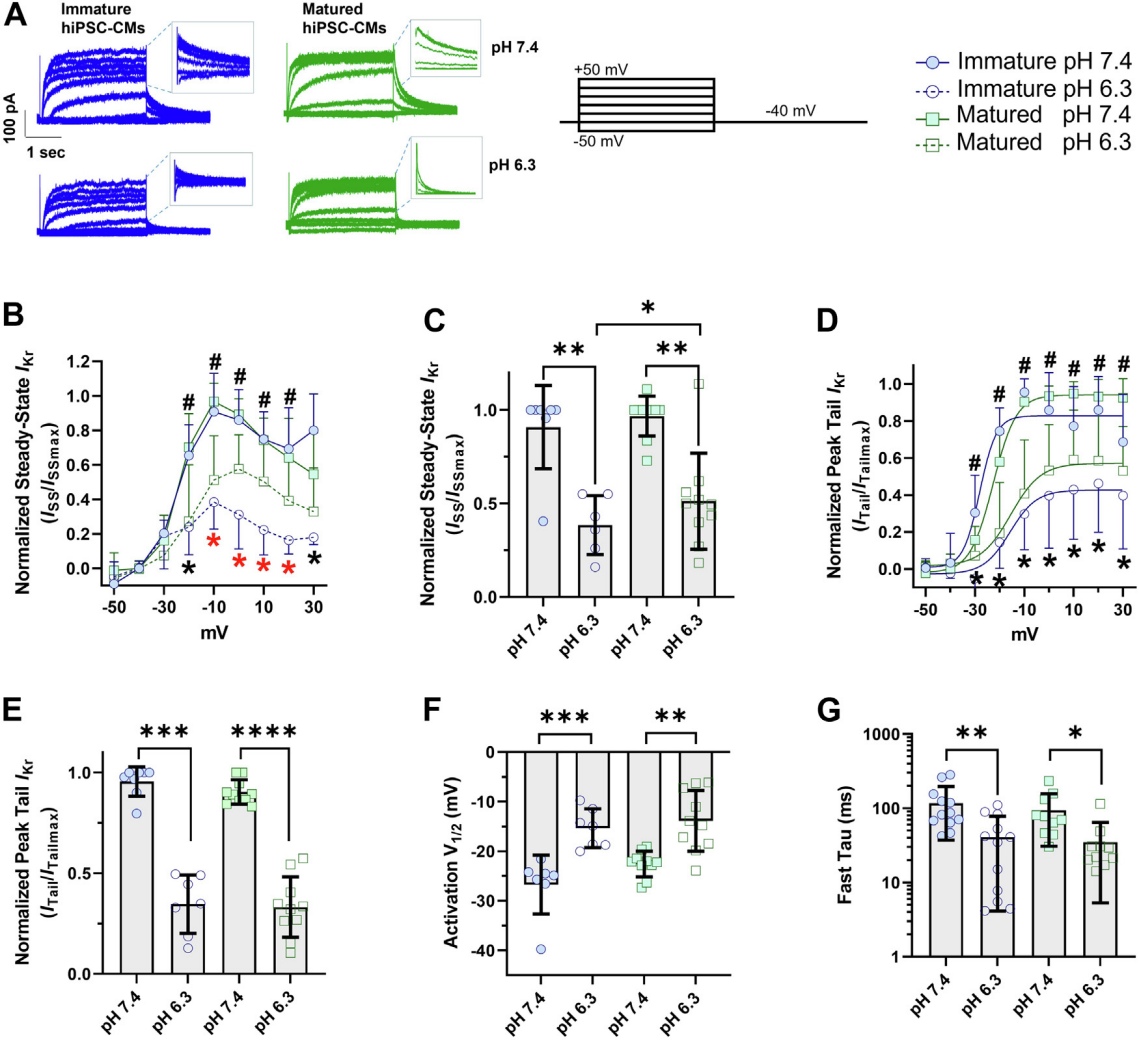
**Proton sensitivity of native *I***_**Kr**_**corresponds with hiPSC-CM maturation.***A*, representative *I*_Kr_ traces elicited by the protocol below from matured and immatured hiPSC-CMs at pH 7.4 and 6.3. *B*, steady-state *I*_Kr_ density in immatured and matured hiPSC-CMs at pH 7.4 and 6.3. *C*, normalized steady-state current densities at −10 mV. *D*, peak-tail *I*_Kr_ density in immatured and matured hiPSC-CMs in control and acidic environment. *E*, normalized peak-tail *I*_Kr_ densities at −10 mV. The symbols ∗ (*black* and *red*) and ^#^ represent the statistical significance of normalized peak tail and steady-state *I*_Kr_ at pH 7.4 *versus* pH 6.3 in immatured and matured hiPSC-CMs, respectively. The symbol ∗ (*red only*) denotes significant difference between immatured and matured hiPSC-CMs at pH 6.3. *F*, voltage dependence of activation (*V*_1/2_) for *I*_Kr_ from immatured and matured hiPSC-CMs in control and acidic environment. *G*, time constants of *I*_Kr_ deactivation recorded from immatured and matured hiPSC-CMs at pH 7.4 and acidic pH 6.3. Data were compared using a two-way ANOVA and a two-tailed Mann–Whitney test. Errors bars represent mean ± SD. N value = 3, n value ≥9. ∗∗∗∗*p* < 0.0001, ∗∗∗*p* = 0.0008, ∗∗*p* = 0.0024, and ∗*p* < 0.05. hiPSC-CM, human-induced pluripotent stem cell–derived cardiomyocyte; *I*_Kr_, rectifier potassium current.

Acidosis has complex electrophysiological effects on hERG1 channels that lead to altered electrical activity. The effects of acidosis on *I*_Kr_ have been studied previously, revealing changes in the voltage dependence of activation when the pH was adjusted from 7.4 to 6.3 (27). Fleet *et al.* (57) reported that acidosis in pig myocardium can drive extracellular pH to as low as pH 6.3. Also, pH 6.3 was previously used to highlight differential proton sensitivity of hERG1a and hERG1b channels in Chinese hamster ovary (CHO) cells (39, 42). Here, we studied the impact of extracellular acidosis on native *I*_Kr_ recorded from either immatured or matured hiPSC-CMs (Table 1 and Fig. 3). Figure 3*A* depicts representative paired *I*_Kr_ traces, recorded first in bath solution titrated to pH 7.4 and then bath solution titrated to pH 6.3. For *I*_Kr_ recorded from either immatured or matured hiPSC-CMs, pH 6.3 decreased the step pulse and tail pulse current density by ∼50% (Table 1 and Fig. 3, *B*–*E*), depolarized the voltage dependence of activation by ∼12 mV (Fig. 3*F*), and dramatically accelerated the time course of deactivation (Fig. 3*G*).

Next, we normalized the magnitude of *I*_Kr_ at pH 6.3 to the maximum *I*_Kr_ magnitude recorded at pH 7.4 for the same cell (Table 1 and Fig. 3, *B*–*E*). Steady-state *I*_Kr_ from immatured hiPSC-CMs was significantly more sensitive to extracellular acidosis than steady-state *I*_Kr_ in matured hiPSC-CMs. Tail *I*_Kr_, however, displayed a similar trend between immatured and PDMS-matured hiPSC-CMs, but it was not statistically significant at −10 mV (*p* = 0.73) (Table 1 and Fig. 3, *B*, *C*, and *E*). Steady-state *I*_Kr_ displayed a roughly twofold increase in inhibition in immatured cells compared with matured cells at 0 through +20 mV (Table 1 and Fig. 3, *B* and *C*). Similar to our initial recordings (Fig. 2*B*), there is a trend that the time course of *I*_Kr_ deactivation recorded from immatured hiPSC-CMs display smaller time constants (117 ± 23 ms at pH 7.4 and 41 ± 11 ms at pH 6.3) than *I*_Kr_ recorded from matured hiPSC-CMs (195 ± 72 ms at pH 7.4 and 80 ± 35 at pH 6.3) (Fig. 3*G*). *I*_Kr_ deactivation at pH 6.3 does not have a slow component of decay.

These results confirm the experimental observations of previous studies on the impact of protons on hERG1 channel activity. And given the distinct deactivation kinetics and proton sensitivities of *I*_Kr_ recorded from immatured *versus* matured hiPSC-CMs, these data also suggest that shifts in hERG1 subunit abundance may mediate the response of native *I*_Kr_ to extracellular acidosis.

### hERG1a and hERG1b expression is dependent upon hiPSC-CM maturation

**Figure 4:**
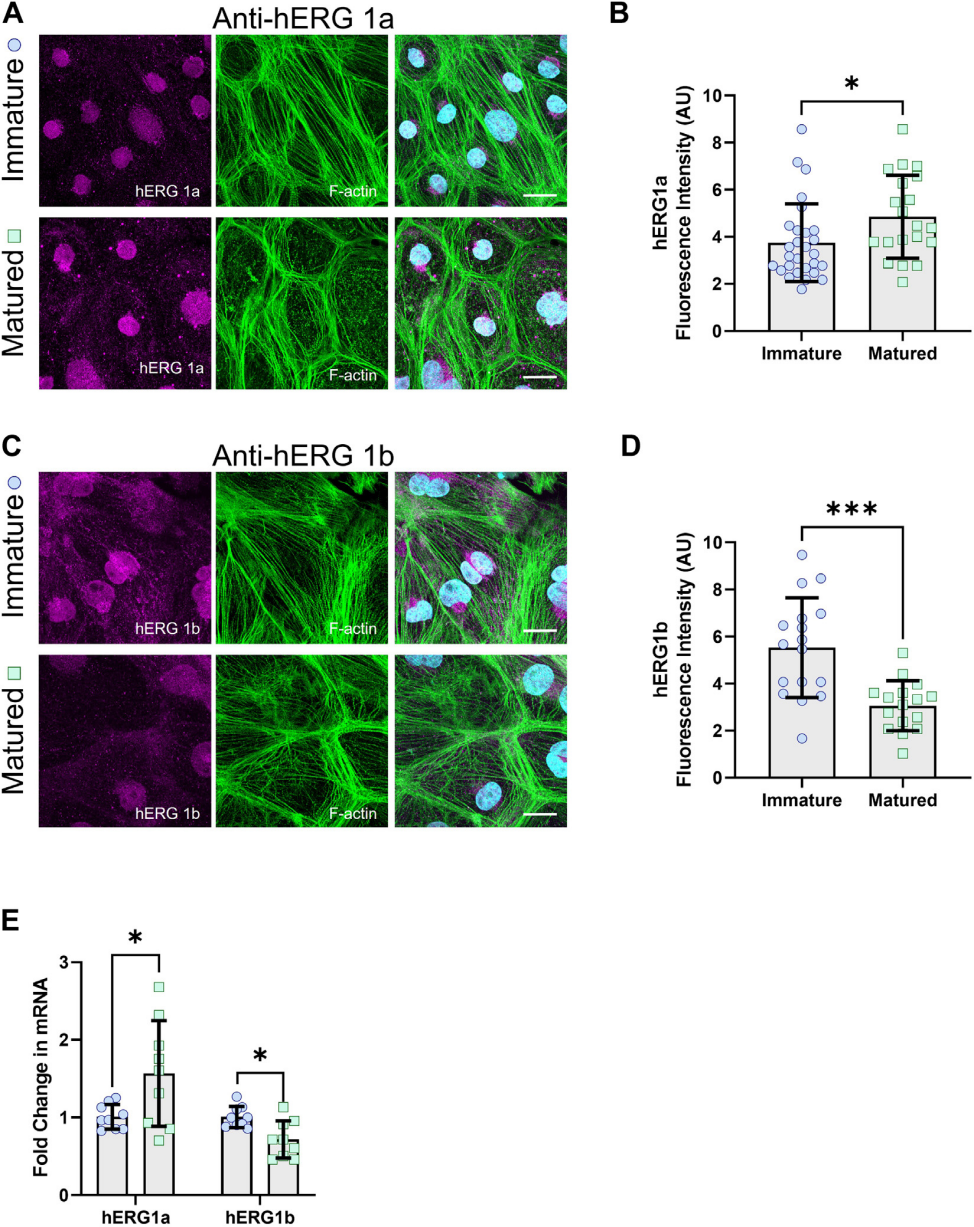
**hERG1 subunit abundance in matured and immatured hiPSC-CMs.***A*, representative immunostainings for hERG1a and F-actin. *B*, quantification of mean hERG1a immunofluorescence from matured and immatured hiPSC-CMs. *C*, representative immunostainings for hERG1b and F-actin. *D*, quantification of mean hERG1b immunofluorescence from matured and immatured hiPSC-CMs. *E*, hERG1a and hERG1b mRNA levels in matured and immatured hiPSC-CMs. Data were compared using a two-tailed Mann–Whitney test. Errors bars represent mean ± SD. N value = 3, n value ≥8. ∗∗∗*p* = 0.0002 and ∗*p* < 0.05. Scale bars indicate 25 μM. hiPSC-CM, human-induced pluripotent stem cell–derived cardiomyocyte.

hERG1 subunit expression is dynamic, varying with development, cell cycle, maturation, and disease states (45, 58, 59, 60, 61, 62). The slowing of *I*_Kr_ deactivation with maturation suggests an increase in hERG1a relative to hERG1b. The diminished proton sensitivity of *I*_Kr_ in matured cells also suggests that hERG1a is upregulated, as hERG1a was shown to be less sensitive to protons, compared with hERG1b, in CHO cells (42). To examine the expression of the hERG1a and hERG1b subunits in matured and immatured hiPSC-CMs, we measured subunit-specific immunofluorescence and mRNA expression levels by quantitative RT–PCR from monolayers cultured on glass or PDMS (Table 2 and Fig. 4).

**Table 2:** hERG1a and hERG1b expression in immatured and matured hiPSC-CMs

Measurement	Immatured hiPSC-CMs	SD	n	Matured hiPSC-CMs	SD	n
hERG1a fluorescence intensity (AU)	3.74^a^	1.65	28	4.85	1.76	20
hERG1b fluorescence intensity (AU)	5.53^b^	2.11	17	3.05	1.06	16
hERG1a mRNA levels (AU)	1.01^a^	0.15	9	1.5	0.67	9
hERG1b mRNA levels (AU)	1^a^	0.13	9	0.71	0.23	9

Two-tailed Mann–Whitney test. N value = 3, n value ≥9.

a *p* < 0.05.

b *p* = 0.0002.

Consistent with our hypothesis, we found that hERG1a immunofluorescence was significantly increased in matured hiPSC-CM monolayers (4.8 ± 0.4 AU) compared with immatured hiPSC-CM monolayers (3.7 ± 0.3 AU) (Table 2 and Fig. 4, *A* and *B*). In contrast, hERG1b immunofluorescence was significantly decreased in matured monolayers (3.0 ± 0.3 AU) compared with immatured monolayers (5.5 ± 0.5 AU) (Table 2 and Fig. 4, *C* and *D*). hERG1a and hERG1b mRNA levels were similarly affected in matured hiPSC-CMs compared with immatured hiPSC-CMs (1.5 ± 0.2-fold change and 0.7 ± 0.07-fold change in matured cells for hERG1a and hERG1b mRNA levels, respectively), as shown in Figure 4*E*. These data demonstrate that hiPSC-CM maturation increases hERG1a expression whereas decreasing hERG1b expression. These data also further support the hypothesis that hERG1 subunit abundance determines *I*_Kr_ proton sensitivity in hiPSC-CMs.

### PAS expression reduces *I*_Kr_ proton sensitivity in immatured hiPSC-CMs

**Figure 5:**
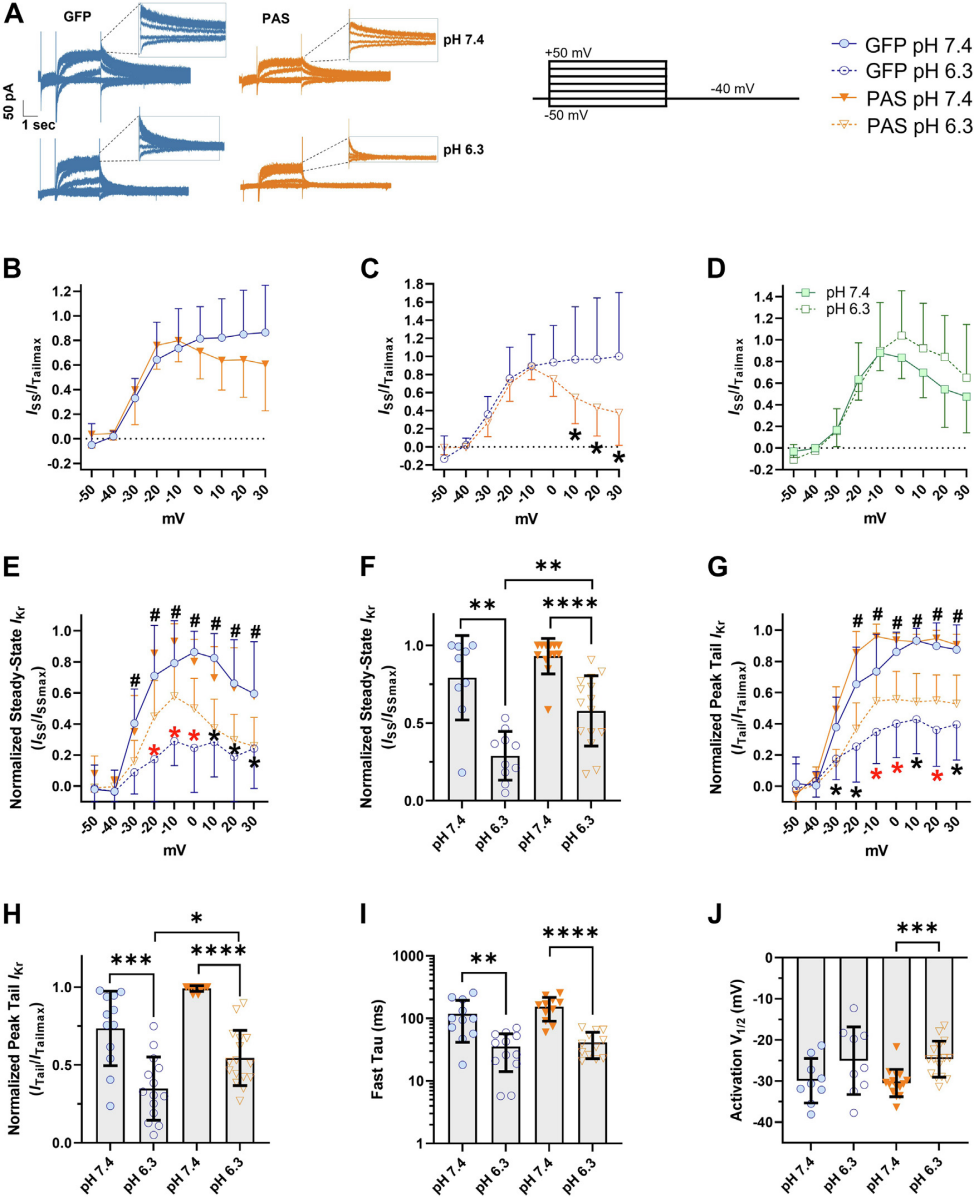
**PAS domain expression diminishes *I***_**Kr**_**proton sensitivity in immatured hiPSC-CMs.***A*, representative *I*_Kr_ traces elicited by the protocol below from PAS (*orange*) and GFP (*blue*)-transduced hiPSC-CMs at pH 7.4 and 6.3. *B* and *C*, steady-state I–V relationships normalized to the maximum peak tail *I*_Kr_ from immatured hiPSC-CMs expressing either GFP or PAS, at pH 7.4 and 6.3, respectively. *D*, steady-state I–V relationships normalized to the maximum peak tail *I*_Kr_ recorded from the same cell for matured hiPSC-CMs at pH 7.4 (*solid symbols*) and 6.3 (*open symbols*), respectively. *E*, steady-state *I*_Kr_ at pH 7.4 and 6.3 in immatured hiPSC-CMs overexpressing PAS or GFP. *F*, normalized steady-state current densities at −10 mV. *G*, peak-tail *I*_Kr_ levels measured at pH 7.4 and 6.3 in immatured hiPSC-CMs overexpressing PAS (*orange*) or GFP (*blue*). *H*, normalized tail current densities at −10 mV. *I*, deactivation kinetics from GFP and PAS-expressing hiPSC-CMs. *J*, voltage dependence of activation (*V*_1/2_) in immatured and matured hiPSC-CMs at pH 7.4 and 6.3. The symbols ∗ (*black* and *red*) and # represent the statistical significance of normalized peak tail and steady-state *I*_Kr_ at pH 7.4 *versus* pH 6.3 in GFP and PAS-transduced hiPSC-CMs, espectively. The symbol ∗ (in *red*) represents statistical differences between PAS and GFP-transduced hiPSC-CMs at pH 6.3. Data were compared using a two-way ANOVA and a two-tailed Mann–Whitney test. Errors bars represent mean ± SD. N value = 3, n value ≥10. ∗∗∗∗*p* < 0.0001, ∗∗∗*p* = 0.0008, ∗∗*p* < 0.0036, and ∗*p* < 0.05. hiPSC-CM, human-induced pluripotent stem cell–derived cardiomyocyte; *I*_Kr_, rectifier potassium current; PAS, Per–Arnt–Sim.

Defining the regulatory elements of hERG1 subunits as they pertain to responses to acidosis is a necessary step toward understanding the functional adaptation and impairment of native cardiomyocytes (CMs) during developmental and pathological processes. Our study revealed that proton inhibition of *I*_Kr_ is enhanced in immatured hiPSC-CMs, where hERG1b expression is upregulated. These data suggest that hERG1b expression promotes proton inhibition of *I*_Kr_. To test this hypothesis, we overexpressed a polypeptide identical to the hERG1a PAS domain in immatured hiPSC-CMs (Table 3 and Fig. 5). This technique has been used in heterologous expression systems (24, 63) and hiPSC-CMs (16) to mask the impact of hERG1b on heteromeric channel gating. When overexpressed, the PAS polypeptide fills the open receptor site left by the abbreviated hERG1b N-terminal domain (24, 63) and thereby transforms heteromeric hERG1a/1b channel gating to a phenotype indistinguishable from homomeric hERG1a channels.

**Table 3:** Biophysical parameters of GFP- and PAS-transduced hiPSC-CMs

Channel parameter		GFP-transduced hiPSC-CMs	SD	*n*	PAS-transduced hiPSC-CMs	SD	n
Steady-state *I*_Kr_ at −10 mV (*I*_SS_/*I*_SSMAX_)	pH 7.4	0.79^a^	0.27	9	0.93^b^	0.11	13
	pH 6.3	0.28^a^	0.15	10	0.57	0.22	15
Peak tail *I*_Kr_ at −10 mV (*I*_Tail_/*I*_TailMAX_)	pH 7.4	0.73^c^	0.23	12	0.99^b^	0.01	13
	pH 6.3	0.34^d^	0.2	14	0.54	0.17	16
Steady-state *I*_Kr_ at −10 mV (pA/pF)	pH 7.4	2.23^d^	2.12	9	2.19^a^	1.75	13
	pH 6.3	1.22	0.84	10	1.49	1.18	15
Peak tail *I*_Kr_ at −10 mV (pA/pF)	pH 7.4	2.18^d^	1.77	12	2.72^d^	2.27	13
	pH 6.3	0.94^d^	0.68	14	1.58	1.12	16
*V*_1/2_ (mV)	pH 7.4	−29.93^c^	5.41	9	−30.49	3.32	15
	pH 6.3	−25.06	8.2	9	−24.68	4.39	15
Fast Tau (ms)	pH 7.4	118.13^a^	76.66	11	153.44^b^	62.86	11
	pH 6.3	35.5	21.36	12	41.42	18.64	12

Two-tailed Mann–Whitney test. N value = 3, n value ≥9.

a *p* < 0.0036.

b *p* < 0.0001.

c *p* = 0.0008.

d *p* < 0.05.

To validate that the PAS polypeptide was appropriately modifying native hERG1 channel function, we first quantified the magnitude of rectification of GFP and PAS-transduced cells. The hERG1a PAS domain promotes inactivation and thereby enhances rectification (20, 24), thus *I*_Kr_ recorded from PAS-transduced cells should display increased rectification. We normalized steady-state currents to the maximum peak tail current recorded from the same cell to quantify the magnitude of current inhibition at positive potentials (rectification). As predicted, PAS-transduced cells displayed enhanced rectification of steady-state currents at both pH 7.4 and 6.3, compared with GFP-transduced controls (Table 3 and Fig. 5, *B* and *C*). These data demonstrate that the overexpressed PAS domain is modifying the function of the extant hERG1 channels at both pH 7.4 and pH 6.3. Remarkably, the degree of rectification observed in immatured hiPSC-CMs expressing PAS was comparable to that seen in matured hiPSC-CMs (Fig. 5*D*).

As expected, pH 6.3 significantly inhibited *I*_Kr_ magnitude (Table 3 and Fig. 5, *A*, *E*–*H*), accelerated *I*_Kr_ deactivation (Table 3 and Fig. 5*I*), and depolarized the voltage dependence of *I*_Kr_ activation (Table 3 and Fig. 5*J*) in both PAS-transduced and GFP-transduced controls. Consistent with our hypothesis, PAS polypeptide overexpression significantly reduced *I*_Kr_ inhibition by protons, compared with GFP controls (Fig. 5, *A*, *E*–*H*). At pH 6.3, normalized steady-state *I*_Kr_ was reduced by only 40 ± 6% in PAS-transduced cells compared with 65 ± 9% in GFP-transduced cells, at −10 mV (Table 3 and Fig. 5, *E* and *F*). Tail *I*_Kr_ was reduced by 44 ± 8% and 54 ± 8% for PAS- and GFP-transduced cells, respectively (Table 3 and Fig. 5, *G* and *H*). In fact, the magnitude of proton inhibition of *I*_Kr_ in PAS-transduced cells was comparable to that observed in our matured hiPSC-CMs (*cf.*, Fig. 3, *B*–*E*). Together, our findings shed light on how the hERG1a PAS domain, in addition to modulating the kinetic properties of channel gating, plays an important role in the response of hERG1 channels to extracellular acidosis. Finally, these data also demonstrate that the relative abundance of hERG1a and hERG1b subunits influences the magnitude of *I*_Kr_ inhibition by extracellular protons.

## Discussion

The present study investigates the impact of extracellular acidosis on native *I*_Kr_ recorded from hiPSC-CMs. First, as demonstrated by others (54), the data presented herein validate that hiPSC-CMs cultured on a soft Matrigel-coated PDMS substrate displayed electrophysiological features consistent with enhanced cardiac maturation, including hyperpolarized RMPs and increased AP amplitude, when compared with cells plated on Matrigel-coated glass. In addition, *I*_Kr_ recorded from PDMS-matured hiPSC-CMs was less sensitive to extracellular protons compared with *I*_Kr_ recorded from immatured hiPSC-CMs. Finally, the decrease in proton sensitivity between immatured and matured cells was mediated by an increase in the relative abundance of hERG1a and hERG1b subunits at the cell surface membrane.

### Proton modulation of hERG1

The impact of external protons on hERG1a has been well described in heterologous expression systems, with distinct effects on single-channel conductance and gating (31, 33, 34, 37, 41). Surprisingly, the impact of protons on native *I*_Kr_ is poorly described. This is of particular importance because native cardiac hERG1 channels comprise both hERG1a and hERG1b subunits (15, 16, 18) and are modulated by other potential accessory subunits (*e.g.*, KCNE1 and KCNE2) and interacting proteins (*e.g.*, KvLQT1) (64, 65, 66, 67).

Here, we demonstrated that reduced extracellular pH inhibited current density and depolarized the voltage dependence of *I*_Kr_ recorded from hiPSC-CMs. These data are consistent with other work on native *I*_Kr_ (27, 68). Interestingly, our study demonstrated that the degree of *I*_Kr_ inhibition by protons correlated with the relative abundance of the hERG1b subunit. Work in CHO cells has also demonstrated that inhibition of hERG1 conductance by protons is more pronounced in channels that contain the hERG1b subunit, compared with hERG1a homomeric channels (42). In this study, proton inhibition of *I*_Kr_ was greatest in immatured hiPSC-CMs, where hERG1b was upregulated. The enhanced inhibition by protons was then abolished by increasing the number of PAS domains per channel, effectively transforming hERG1b subunits into hERG1a subunits. Importantly, the time course of deactivation is normally a reliable marker of PAS activity, where PAS-deficient heteromeric hERG1a/1b channels display faster deactivation compared with homomeric hERG1a channels. However, because of the dramatic accelerating effects of protons on deactivation, it is not a reliable marker of PAS action at reduced pH. Overall, our data demonstrate that hERG1 subunit stoichiometry mediates proton inhibition of *I*_Kr_ in hiPSC-CMs.

**Figure 6:**
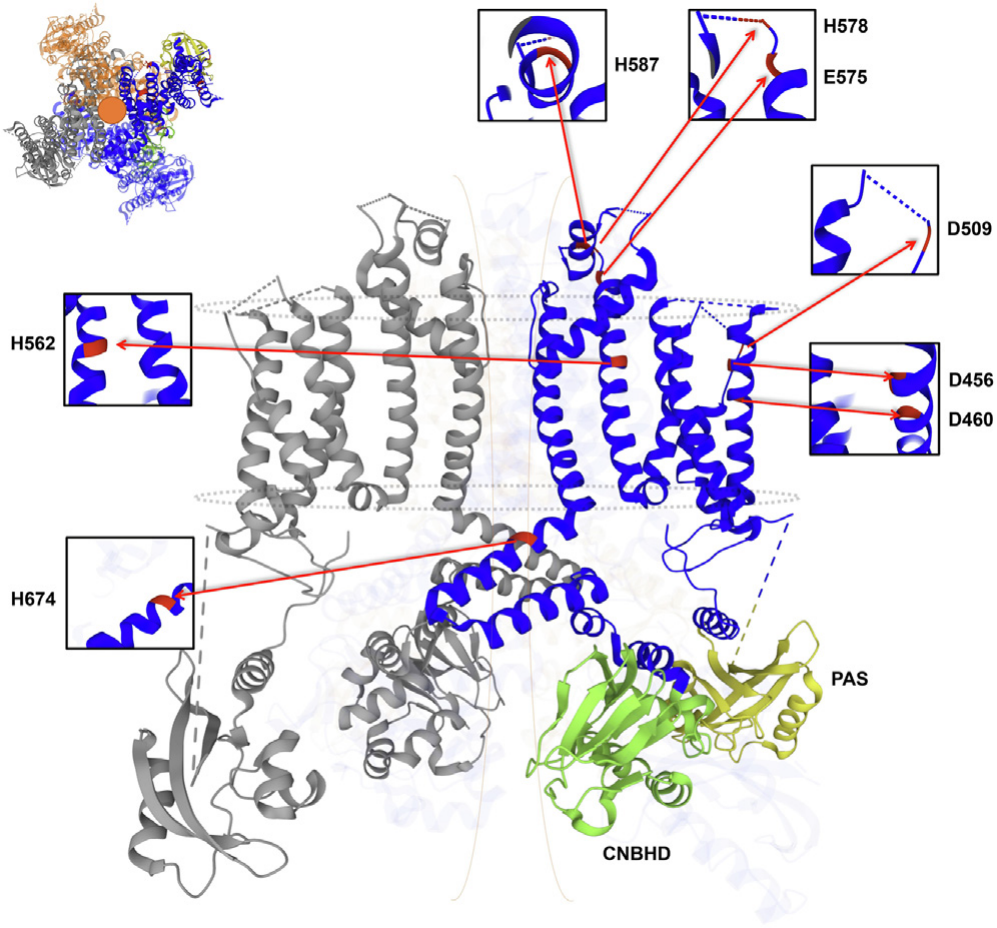
**Cryo-EM structure of the hERG1a channel with predicted locations of residues identified as proton sensors.** Predicted pronatable amino acids modeled on the hERG1a cryo-EM structure (Protein Data Bank ID: 5VA1) using the Research Collaboratory for Structural Bioinformatics Protein Data Bank (rcsb.org) (33, 34, 41, 69, 70, 99). Eight residues are highlighted: E575, H578, and H587 in the pore turret, D509 in the S3 helix, D456 and D460 in the S2 helix, H562 in the S5 helix, and H674 in the S6 helix–CNBHD linker. *Black boxes* depict expanded view of each residue position. The CNBHD and PAS are highlighted in *green* and *yellow*, respectively. *Dashed blue lines* represent regions unresolved in the cryo-EM structure. *Inset* depicts a top–down view of the tetrameric subunit arrangement of the hERG1a channel cryo-EM structure. cyclic nucleotide–binding homology domain; PAS, Per–Arnt–Sim.

It is unclear how hERG1b selectively enhances proton inhibition of channel conductance without altering the impact of protons on channel gating. This is somewhat surprising given the pronounced accelerating effects that hERG1b has on hERG1 channel gating, particularly deactivation (14, 15, 20). hERG1 has proton-binding sites at the pore and voltage-sensing domains that modulate conductance and gating, respectively, with different pH sensitivities (Fig. 6) (31, 33, 34, 37, 41, 69). At the voltage-sensing domain, mutating a trio of aspartates to alanines (D456A/D460A/D509A) disrupts proton modulation of channel gating (33, 69, 70). Proton block, however, is critically dependent upon residues E575 and H578 at the hERG1 pore turret, where the combined mutations E575Q and H578N abolish proton block without affecting proton modulation of deactivation (41). Although they are located on the outer circumference of the hERG1 pore, these proton-binding sites (at least E575 and H578) alter the electrostatic environments in and around the selectivity filter (41). It was proposed that the outer hERG pore near the selectivity filter is somewhat flexible (71, 72, 73), underlying inactivation and possibly providing a mechanism to transmit protonation of E575 and H578 to changes in hERG1 channel conductance and open time (41, 72, 74). Nonetheless, this must be approached with caution because only the E575 side chain was shown in the hERG1 cryo-EM structure to directly interact with residues that connect to the selectivity filter (72). Because the residues involved in proton sensitivity are found in both hERG1a and hERG1b, it is possible that based on the cryo-EM structure, the greater effect we observed on cells preferentially expressing hERG1b was due to indirect/allosteric consequences of the unique hERG1b N terminus that favor exposure of E575 and H578 to protons. Another possibility is that the residues H578 and H587, which are found in a relatively disordered channel region (Fig. 6) (72), may be involved in the removal of the proton block of the pore because they are in a more flexible region of the channel and may interact with other residues (*e.g.*, D580) that can modulate the channel's proton sensitivity indirectly, adding to the possibility of distinct conformations during gating and/or channel composition. Finally, intracellular acidosis does not affect hERG1a homomeric channels (38), but we cannot rule out that the short hERG1b N terminus may expose intracellular proton-binding sites, otherwise occluded by the hERG1a PAS domain.

### Protons in *I*_Kr_-mediated cardiac dysfunction

Cardiac acidosis occurs under a number of physiological and pathophysiological conditions. Two conditions, ischemic heart disease and SIDS, are particularly affiliated with hERG1 dysfunction. In chronic cardiac dysfunction, that is, heart failure, native *I*_Kr_ is significantly downregulated (75, 76) and the relative abundance of hERG1b to hERG1a is increased (51). These changes in *I*_Kr_ occur alongside the downregulation of other major K^+^ currents: *I*_Ks_, *I*_to_, and *I*_K1_ (77, 78, 79). The reduced I_K_ contributes collectively to the reduced repolarization reserve, prolonged AP duration, and overall heightened arrhythmogenic potential in the failing myocardium. Our data suggest that a relative increase in hERG1b in the failing heart would also enhance *I*_Kr_ sensitivity to protons during ischemic events. And although hERG1b homomeric channels may not exist in adult hearts—hERG1b subunits preferentially associate with hERG1a (80, 81)—the relative expression of hERG1a and hERG1b subunits appears heterogeneous in cardiac tissue (17, 48, 82). In this regard, regional variation in hERG1 isoform abundances could facilitate heterogeneity of repolarization and arrhythmogenesis during acidosis.

### hERG1 subunits in neonatal and fetal demise

*KCNH2* variants have long been linked with SIDS (3, 7, 13). Respiratory acidosis is one hypothesis proposed to explain the association of stomach sleeping with SIDS (8, 83). Interestingly, hERG1a mRNA is upregulated and hERG1b mRNA is downregulated in adult human cardiac tissue compared with fetal cardiac tissue (3). These molecular data combined with our electrophysiological data suggest that upregulated hERG1b in the immature heart could promote proton inhibition of *I*_Kr_ during respiratory acidosis and thereby contribute to SIDS. The shifts in subunit abundance during maturation also predict that the pathophysiological impact of hERG1b-specific mutations would be greatest in the immature heart and vice versa for hERG1a-specific mutations. Indeed, the only two hERG1b-specific mutations identified to date were a case of intrauterine fetal death, R25W (3), and an 8-year old girl, A8V (20). Interestingly, similarly to other mutations found in SIDS cases (R273Q and R954C/K897T), the mutation R25W generates a profound reduction in current density when expressed as heterotetramers with the hERG1a subunit (3, 4, 9).

For normal heart function, these two hERG1 subunits must be functionally expressed. Changes in the abundance of hERG1a or hERG1b can cause proarrhythmic events (16, 20, 46). Clearly, there is a link between LQTS2 and intrauterine fetal death (3, 84, 85, 86). hERG1 channel variants that have been solely linked with SIDS have the potential to be LQTS variants. And it is possible that LQTS cases are being disguised under the SIDS umbrella, setting a precedent for future research into the role of cardiac channelopathies.

### Subunit-selective modulators

Protons are not the only factor shown to differentially modulate homomeric and heteromeric hERG1 channels. Several studies have demonstrated that subunit abundance, and its impact on gating, mediates the channel’s response to a subset of clinically relevant drugs (20, 87, 88). In addition, atrial natriuretic peptide and cGMP perfusion were both shown to selectively inhibit hERG1b-containing channels heterologously expressed in human embryonic kidney 293 cells (89). In the same study, the authors demonstrated that cGMP inhibited *I*_Kr_ recorded from atrial but not ventricular murine myocytes, suggesting that mERG1b expression was greater in atrial tissue than in ventricular murine tissue (89). Assuming that mERG1b is primarily expressed in the atria, and based on findings from computational modeling indicating that the gain-of-function mutations L532P and N588K cause a higher and earlier peak of *I*_Kr_ during atrial APs and lead to rotor formation (90), we postulate that ERG1b subunit expression may play a key role in atrial fibrillation (91). In contrast, hERG1b shows a protective effect against oxidative inhibition, presumably by regulating access to a key residue in the channel’s C-linker domain C723 (hERG1a numbering). Roughly, two-thirds of the protective effect from hERG1b were attributable to the subunit’s acceleration of channel deactivation (92).

The fact that hERG1b is upregulated in “immatured” hiPSC-CMs underscores the need for increased understanding of mechanisms regulating hERG1 subunit abundance. Drugs that preferentially target hERG1 isoforms may be one approach to overcome obstacles in treating disorders in the heart and other tissues where hERG1 is a contributing factor. The two hERG1 isoforms are expressed in distinct ratios and contribute differently to the maintenance of hERG1 currents in tissues where hERG1 is functional. For example, while hERG1b is expressed at lower levels in the human heart, it is the predominant isoform in tumor cells (93). In B-cells and T-cell lineage, hERG1b is upregulated, whereas the other isoform, hERG1a, is downregulated (94). Therefore, identifying the mechanisms that control hERG1 subunit abundance could improve clinical therapies in diseases throughout the body.

## Conclusion

The experimental data presented herein show for the first time the effects of extracellular acidosis on native *I*_Kr_ recorded from hiPSC-CMs following shifts in hERG1 subunit abundance. And although the exact tetrameric conformation of native hERG1 channels remains elusive, these findings provide insight into the response of adult and immatured CMs to an acidic environment.

### Limitations

Here, we report data from experiments conducted in immatured and matured hiPSC-CMs. While our studies demonstrate the impact of extracellular acidosis in a human CM model, hiPSC-CMs still cannot recapitulate the chamber-specific or layer-specific electrical phenotypes of intact cardiac tissue. And though tools to enhance hiPSC-CM maturation have improved, the “PDMS-matured” hiPSC-CMs used in this study still display significant markers of immaturity, including irregular shape, disorganized contractile machinery, and spontaneous AP firing. Thus, the PDMS-matured hiPSC-CMs are not an accurate model of an adult ventricular CM, and comparison of the effects observed in this article with the adult myocardium should be done with caution. In addition, native *I*_Kr_ magnitudes are relatively small, particularly at pH 6.3, which increases experimental variability. Nonetheless, these data provide important insight into the triggers of *I*_Kr_ dysfunction during extracellular acidosis.

## Experimental procedures

### Stem cell culture and cardiac differentiation

DF19-9-11 human iPS cells were obtained from the WiCell Research Institute. Cells were cultured and differentiated into CMs using the GiWi protocol, as described (95). Stem cells were seeded on Matrigel-coated plasticware with iPS-brew medium. Spontaneous differentiation was removed, and cells were passed at 70% confluence. At the day of cell passage, cells were reseeded to continue the line or to grow monolayers for cardiac-directed differentiation. About 4 × 10^5^ cells were plated into each well of a 6-well plate and cultured to ∼80% confluence for treatment with glycogen synthase kinase 3 inhibitor and induction of mesodermal differentiation (day 0). Following mesodermal differentiation, cells were treated with a Wnt inhibitor for induction of cardiac mesoderm (day 2). On day 4, Wnt inhibitor was removed to direct the cells into cardiac progenitor cells. hiPSC-CMs with autonomous contractility emerged 8 to 10 days after initiation of cardiac-directed differentiation. The hiPSC-CMs were cultured until 20 days after initiation of differentiation and purified using by magnetic bead–assisted isolation with an hiPSC-Derived Cardiomyocyte Isolation Kit, human (Miltenyi Biotec). Purified hiPSC-CMs were then plated on either Matrigel-coated glass coverslips (immatured cells) or Matrigel-coated PDMS (matured cells) for 7 days before completing experiments. hiPSC-CMs cultured on glass display electrical characteristics consistent with embryonic/early fetal CMs including depolarized RMP, reduced AP upstroke velocity, and reduced AP amplitude (96, 97). hiPSC-CMs cultured on PDMS display characteristics consistent with enhanced maturation, similar to late fetal/neonatal electrophysiology including: hyperpolarized RMPs, increased AP amplitude and upstroke velocity, increased expression of mature sarcolemma components (*e.g.*, SCN5A, Kir2.1, and Cx43), as well as myofilament markers (cTnI), and faster conduction velocities (54, 56).

### Adenovirus transduction of hiPSC-CMs

hiPSC-CMs were replated at a low density onto Matrigel-coated glass coverslips in a 6-well plate and maintained at 37 °C in 5% CO_2_ for at least 72 h before transduction. hiPSC-CMs were then transduced for 48 h and then refreshed with RPMI/B27+ media. hiPSC-CMs were transduced with either GFP-encoded adenovirus or the scFv2.10-CFP-encoded adenovirus (University of Michigan Viral Vector Core). Fluorescence was monitored after 24 h to verify successful transduction. Contracting fluorescent hiPSC-CMs were used for electrophysiology experiments 48 h after transduction.

### Quantitative RT–PCR

For quantitative evaluation of the steady-state mRNA expression in hiPSC-CM cultures, total RNA was prepared using the RNeasy Mini Kit (Qiagen), including DNAse treatment. About 300 ng of RNA were reverse transcribed and converted to complementary DNA with oligo(dT)_12–18_ primers using reverse transcriptase according to the manufacturer's specifications, M-MLV Reverse transcriptase (catalog no.: 28025-013; Invitrogen). Quantitative PCR was performed using IDT Mastermix (catalog no.: 1055772; Thermo Fisher) and TaqMan assay primers (catalog nos.: 4331182 and 43513752, 10 μM; Thermo Fisher) for *KCNH2*, *KCNH*1a, and *KCNH*1b isoforms. The PCR condition consisted of 95 °C for 30 s, followed by 39 cycles of 95 °C for 3 s and 60 °C for 20 s, followed by melting-curve analysis to verify the correctness of the amplicon.

The samples were analyzed in technical triplicates using the primers included in the TaqMan Assay system (Invitrogen) and run in a Bio-Rad C1000 Touch Thermal Cycle CFX96 (Applied Biosystems). The expression of the mRNA of the gene of interest relative to the internal control GAPDH in samples from immatured and matured hiPSC-CMs was calculated by the ΔΔCT method, based on the threshold cycle (CT), as fold change = 2^∧^−(ΔΔCT), where ΔCT = CT_gene of interest_ − CT_GAPDH_ and ΔΔCT = ΔCT_Matured hiPSC-CMs_ – ΔCT_Immatured hiPSC-CMs._ From each experiment, the complementary DNA of three cell culture wells was measured as biological replicates of each cell maturation state. Each cell culture well was measured from at least three separate CM differentiation.

### Immunocytochemistry

hiPSC-CMs were seeded either on glass or PDMS and fixed with 4% paraformaldehyde/PBS for 15 min. Then, hiPSC-CMs were washed 5 min with PBS and blocked with block solution (PBS + 1% bovine serum albumin + 0.5% Triton-X + 10% goat serum secondary antibodies) for 1 h. Incubation with primary antibodies was done in block solution for overnight at 4 °C. The next day, to washout the excess of primary antibody (Ab), hiPSC-CMs were washed 3× for 5 min with PBS. Next, secondary antibodies in block solution (without Triton-X) were added to each slip and incubated for 1 h in the dark at room temperature. hiPSC-CMs were kept in dark, washed with PBS 3× for 5 min, and mounted with ProLong Gold antifade reagent (Thermo Fisher) and a coverslip. Both primary and secondary antibodies were diluted in block solution (without Triton-X).

Differentiated cardiac lines were validated using immunocytochemistry targeting actin (phalloidin; catalog no.: A12379; Thermo Fisher) to display the cardiac sarcomeric organization and patch clamp electrophysiology measuring cardiac *I*_Kr_, indicative of hERG1 expression. Phalloidin-488 comes with a fluorophore conjugated; so no secondary Ab incubation was needed. To target the hERG1a isoform, hiPSC-CMs were immunolabeled with a 1:200 dilution of the primary Ab (catalog no.: ALX-215-050-R100; Enzo Life Sciences). To target the hERG1b isoform, the primary Ab (catalog no.: ALX-215-051-R100) was used in a 1:200 dilution (Enzo Life Sciences). In both cases, a 1:250 dilution of secondary Ab goat anti-rabbit Alexa Fluor 647 (catalog no.: 4050-31; Southern Biotec) was used. The nuclei were labeled using 1:1000 dilution of 4′,6-diamidino-2-phenylindole (1 μg/ml) for 15 min (Thermo Scientific; catalog no.: 62248). Immunostained preparations were analyzed by confocal microscopy, using a confocal microscope (Zeiss 880) to determine protein localization. Images were analyzed using FIJI where we measured mean hERG immunofluorescence intensity in the cytoplasmic region of the cell from matured or immatured hiPSC-CMs.

### Electrophysiology

Standard patch-clamp techniques were used to measure both AP clamp waveform and *I*_Kr_. All recordings were completed at physiological temperature (37 ± 1 °C) using whole-cell patch clamp with an IPA Integrated Patch Amplifier run by SutterPatch (Sutter Instrument) and Igor Pro 8 (Wavemetrics). Leak subtraction was performed offline based on measured current observed at potentials negative to *I*_Kr_ activation. The interpulse duration for all recordings was 10 s where cells were at −40 mV.

Data were sampled at 5 kHz and low-pass filtered at 1 kHz. Cells were perfused with extracellular solution containing (in millimolar): 150 NaCl, 5.4 KCl, 1.8 CaCl_2_, 1 MgCl_2_, 15 glucose, 10 Hepes, 1 sodium pyruvate, and titrated to pH 6.3 and 7.4 using NaOH. Fleet *et al.* (57) reported that acidosis in pig myocardium can drive extracellular pH to as low as pH 6.3. Also, pH 6.3 was previously used to highlight differential proton sensitivity of hERG1a and hERG1b channels in CHO cells (39, 42). Recording pipettes had resistances of 2 to 5 MΩ when backfilled with intracellular solution containing (in millimolar): 5 NaCl, 150 KCl, 2 CaCl_2_, 5 EGTA, 10 Hepes, 5 MgATP, and titrated to pH 7.2 using KOH. Intracellular solution aliquots were kept frozen until the day of recording. We kept the intracellular solution on ice during recordings and discarded it 2 to 3 h post-thaw.

To isolate *I*_Kr_, all protocols were completed before and after extracellular perfusion of 2 μM of the *I*_Kr_-specific blocker, E-4031. To inactivate sodium currents, a 100-ms step to −40 mV was applied before any *I*_Kr_ recordings. To assess the voltage dependence of *I*_Kr_ activation, cells were stepped from a holding potential of −40 mV to a 3 s prepulse between −50 and +50 mV in 10 mV increments. Tail currents were then measured during a −40 mV, 3 s test pulse. Peak tail current was normalized to cellular capacitance, plotted as a function of prepulse potential, and fitted with the following Boltzmann equation:

(1) $$y=\left[\frac{A_{1}-A_{2}}{1+e^{\left({V-V}_{0}\right)/k}}\right]+A_{2,}$$

where *A*_1_ and *A*_2_ represent the maximum and minimums of the fit, respectively, *V* is the membrane potential, *V*_0_ is the midpoint, and *k* is the slope factor. The time course of *I*_Kr_ deactivation was assessed by fitting current decay during the test pulse with a double exponential function:

(2) $$y=Y_{0}+A_{1}e^{-t/\tau_{1}}\;A_{2}e^{-t/\tau_{2}}\text{,}$$

where *Y*_0_ is the asymptote, *A*_1_ and *A*_2_ are the relative components of the fast and slow time constants τ1 and τ2, respectively. The magnitude of *I*_Kr_ rectification was quantified by dividing the average *I*_Kr_ during the final 10 ms of each step pulse by the maximum peak outward tail current evoked at −40 mV. Repolarizing charge was calculated by integrating *I*_Kr_ recorded during a voltage protocol that mimics a human ventricular AP (98).

### Statistical analysis

Analysis was completed using Prism 8 (GraphPad Software, Inc) and Igor Pro 8. Values were first tested for normality (Shapiro–Wilk test) and for outlier identification (ROUT and Grubbs’ tests) before statistical evaluation. All data are reported as mean ± SD and were compared using a nonparametric Mann–Whitney test or two-way ANOVA with a Bonferroni post hoc test, where applicable. Statistical significance was taken at *p* < 0.05. Data points greater than two times the SD were termed outliers and excluded from analysis. The fraction of excluded data was no more than the 10% of each dataset. Unless stated otherwise, the number *n* of observations indicated reflects the number of hiPSC-CMs recorded from each cell line from at least three differentiations. All experiments were performed as a single-blind study to avoid sources of bias.

## Data availability

All data generated or analyzed in this study are included in this article.

## Conflict of interest

The authors declare that they have no conflicts of interest with the contents of this article.
